# Renal Disease in Cats and Dogs—Lessons Learned from Text-Mined Trends in Humans

**DOI:** 10.3390/ani14233349

**Published:** 2024-11-21

**Authors:** Christos Dadousis, Anthony D. Whetton, Kennedy Mwacalimba, Alexandre Merlo, Andrea Wright, Nophar Geifman

**Affiliations:** 1 School of Health Sciences, Faculty of Health and Medical Sciences, University of Surrey, Guildford GU2 7XH, UK; n.geifman@surrey.ac.uk; 2 Veterinary Health Innovation Engine, School of Veterinary Medicine, University of Surrey, Guildford GU2 7XH, UK; a.whetton@surrey.ac.uk; 3 School of Biosciences and Medicine, Faculty of Health and Medical Sciences, University of Surrey, Guildford GU2 7XH, UK; 4 Zoetis, Inc., Parsippany, NJ 07054, USA; kennedy.mwacalimba@zoetis.com (K.M.); alexandre.merlo@zoetis.com (A.M.); andrea.wright@zoetis.com (A.W.)

**Keywords:** renal disease, kidney failure, chronic insufficiency, cat, dog, human, comparative analysis

## Abstract

Chronic kidney disease (CKD) is characterised by progressive kidney damage and encompasses a broad range of renal pathologies and aetiologies. In humans, CKD is an increasing global health problem, in particular in the western world, while in cats and dogs, CKD is one of the leading causes of mortality and morbidity. Here, we aimed to develop an enhanced understanding of the knowledge base related to the pathophysiology of renal disease and CKD in cats and dogs. To achieve this, we leveraged a text-mining approach for reviewing trends in the literature and compared the findings to evidence collected from publications related to CKD in humans. Applying a quantitative text-mining technique, we examined data on clinical signs, diseases, clinical and lab methods, cell types, cytokine, and tissue associations (co-occurrences) captured in PubMed biomedical literature. Further, we examined different types of pain within human CKD-related publications, as publications on this topic are sparser in companion animals, but with the growing importance of animal welfare and quality of life, it is an area of interest. Our findings could serve as substance for future research studies. The systematic automated review of relevant literature, along with comparative analysis, has the potential to summarise scientific evidence and trends in a quick, easy, and cost-effective way. Using this approach, we identified targeted and novel areas of investigation for renal disease in cats and dogs.

## Simple Summary

Chronic kidney disease (CKD), defined by the presence of damage or progressive deterioration of the function of one or both kidneys, is an increasing global health problem in humans, in particular in the western world, while in cats and dogs, CKD is one of the leading causes of mortality and morbidity. However, although there is extensive scientific literature on CKD in humans, in veterinary medicine, this is limited. We applied a computational approach to capturing knowledge in the biomedical literature and conducted a comparative analysis of trends between humans and cats and dogs. Using this approach, we identified novel areas of investigation for renal disease in cats and dogs and emphasise the need to address CKD-related pain.

## 1. Introduction

Chronic kidney disease (CKD) refers to abnormality in one or both kidneys that is present for an extended period (at least three months) [1,2]. It is a common medical condition that appears in humans and companion animals, such as cats and dogs, with prevalence rates depending on many factors (e.g., sex, age, breed, and species) and varying from ~11 to 13% in humans [3] (age dependent [3]) and between 0.5 and 3.7% in dogs [4,5,6,7]. Though the prevalence is lower in younger cats (2–4% in cats < 10 years old [8,9,10]), in the last decade, CKD has been considered as a major issue [11] in elderly cats (>10 years old) and remains a leading cause of mortality and morbidity, with prevalence of up to 30–40% [10,12]. In humans, in recent years, CKD has evolved into a global health concern [13,14], especially in the western world, with the annual number of deaths globally directly caused by CKD estimated to be 5–10 million [15]. In England, it is projected that >4 million people (>16 years old) will have CKD stage 3–5 (moderate–severe) by 2036 [16]. The increase in CKD prevalence in humans is closely related to the increased ageing of the population but also to the increase in other medical conditions (comorbidities), such as obesity, type 2 diabetes, cardiovascular disease, and hypertension [17,18,19].

CKD is highly heterogeneous, with diverse causes, aetiologies, and underlying pathologies, including diabetes, hypertension, glomerular diseases, and genetic factors, among others, which can lead over time to kidney and cardiovascular disease and renal dysfunction, depending on the species involved [2,14]. As a result, individual variability exists, making diagnosis and treatment challenging. For instance, although initially it was suggested that acute kidney injury (AKI) and CKD should be diagnostically discriminated in dogs and cats [2], it has recently been proposed that one can lead to the other, similar to cases in humans [20,21].

The guidelines of the International Renal Interest Society (IRIS, http://www.iris-kidney.com/guidelines/guidelines_updates_2023.html; accessed on 28 February 2024) for diagnosing CKD in cats and dogs include clinical signs and physical examination. In brief, the diagnosis of stage 1 and early stage 2 CKD is based on any of the following: a persistent increase in creatinine, renal proteinuria, serum creatinine, or symmetric dimethylarginine (SDMA), with a suggested threshold of SDMA > 14 μg/dL; and abnormal kidney imaging or shape noticed at surgery. SDMA is considered as a surrogate marker of the glomerular filtration rate (GFR). Other factors such as breed, sex, age, diet, and medication history are suggested to be considered for the diagnosis of CKD. For example, cats with CKD lose body weight, which in turn leads to increased morbidity and mortality [22]; thus, nutritional modification and the use of renal diets (mainly restrictions on phosphorus and protein) play a significant role in CKD therapy [23,24]. Currently, in cats, the gold standard to detect impaired renal function is via the GFR, but this is not tractable for or used in routine veterinary practice [25]. Several blood and urine biomarkers exist that are associated with CKD and can be used for predicting the development of CKD in cats [2,25]. Existing biomarkers in cats are generally grouped into (i) systemic GFR biomarkers (e.g., cystatin C, symmetric dimethylarginine), (ii) biomarkers of metabolic derangement (fibroblast growth factor-23), (iii) biomarkers of kidney stress or injury (e.g., albumin, transferrin, neutrophil gelatinase-associated lipocalin, liver-type fatty acid-binding protein, kidney injury molecule-1, vascular endothelial growth factor, urinary cystatin C, heat shock protein-72, and F2-isoprostanes), and (iv) biomarkers of renal fibrosis (e.g., transforming growth factor-β1, procollagen type III amino-terminal propeptide) [25]. However, to date, an accurate biomarker for routine applications in clinical laboratories does not exist [26]. In dogs, an early prediction statistical model for CKD was recently proposed for routine clinical tests, utilising electronic health records from ~60 k dogs in the USA [27]. The proposed machine learning model was based on the age and weight of dogs and on records of urine specific gravity, urine protein, creatinine, and blood urea nitrogen. In humans, CKD is primarily diagnosed using GFR or estimated GFR (eGFR) or urine creatinine levels [21,27], with recent advancements made in the identification of blood biomarkers for disease progression [28]. Moreover, although in human medicine, eGFR is deployed in clinical settings and routinely applied to track the progression of CKD patients, in companion animals, a validated eGFR model does not exist [27].

Compared to dogs, cats with CKD have, in general, better prognosis and longer survival times [29,30]. In dogs with CKD, there is evidence that better body condition score is positively associated with better prognoses and longer survival [23]. The “obesity paradox”, referring to the better survival of overweight or obese people with CKD compared with normal- or under-weight people [23], is well documented in humans, albeit not well understood. Similarly, dogs with high body condition score (7–9, at a scale of 1–9) considered as overweight at the time of CKD diagnosis were positively associated with improved survival [23]. Early-stage CKD is most commonly silent, and thereby, the disease can remain undetected, allowing for its progression, which presents challenges for the clinician in both cats and dogs. Further, some clinical signs that may be informative can be hard to assess in companion animals (e.g., bone or joint pain) or are not yet known to be associated with the disease in cats and dogs (e.g., fatigue, anaemia, and oral ulcer). At advanced stages of CKD when clinical symptoms are evident, patients are already at high risk of cardiovascular-related mortality, with no available treatment to date [14].

We hypothesise that while the pathophysiology may differ across and within species, important similarities may exist, and the examination of these could provide new insights into novel signs and markers for early disease detection and novel therapeutic approaches.

Published scientific research provides a rich source of disease-related knowledge. Many disease-related phenotypic trends are captured in the biomedical literature and can be extracted from freely available PubMed records. However, the vast amount of available literature on CKD makes review time-consuming and labour-intensive. To overcome these limitations, text mining offers an appropriate tool to search and summarise results from the literature [31,32] and enable the close targeting of future research efforts. Such targeting is especially relevant in companion animal research given the 3Rs initiative to refine, reduce, or replace the use of animals in laboratory settings.

An in-depth review of the existing literature to extract relevant links and associations, coupled with a comparative analysis across humans and companion animals (hereafter the term “companion animals” specifically refers to cats and dogs), offers an opportunity to identify common as well as unique characteristics of renal disease and CKD in different species to gain a better understanding of the disease in species where scientific evidence is limited. A variety of text-mining techniques and tools exist for extracting information from PubMed records (abstracts and full articles) [31,32]. Here, we leverage a tool developed to capture trends and associations from Medical Subject Headings (MeSH—the National Library of Medicine’s controlled vocabulary thesaurus used for indexing articles for PubMed) associated with PubMed entries. This tool’s utility has been demonstrated in identifying trends related to immune mechanisms and disease [33], coronaviruses and related respiratory infections [34], as well as digital health advancements in the UK [35].

In keeping with the concept of One Health, the aim of this study was to identify and compare published evidence on associations of different MeSH terms with CKD and renal disease in cats, dogs, and humans, with a focus in clinical signs and manifestations.

## 2. Materials and Methods

### 2.1. PubMed Data

Three corpuses (datasets) of PubMed records were captured, one for human-related publications, one for cats, and a third for dogs. The PubMed database, having the most complete journal coverage in health, biomedicine, and veterinary medicine, was considered sufficient for our analysis. For the human-related corpus of PubMed records, PubMed was searched for any records related to chronic renal insufficiencies OR chronic renal insufficiency OR Kidney Failure, Chronic AND Homo Sapiens; for cats and dogs, the same search was carried out but also included the term “Kidney Disease” and the appropriate species term (“felis catus” or “canis familiaris”, respectively). The term “Kidney Disease” was only included for the cat and dog corpuses to ensure a sufficient number of publications were available for the mining of potential associations. In humans, the term “Chronic Kidney Disease” was sufficient to capture a large enough corpus of publications that were relevant and of focus for the current study. The list of search strings used to query the PubMed database for renal disease and CKD-related publications is presented in Table 1. The PubMed database was accessed on the 10 April 2023.

### 2.2. Text Mining

The approach to mine PubMed records for MeSH associations has been previously described by Geifman et al. (2016) [33] and by Geifman and Whetton (2020) [34]. Here, we applied this methodology to search for associations with different diseases, diagnostic and research methods, cytokines, cell types, tissue types, as well as CKD-related terminology (clinical signs) in cats, dogs, and humans. In brief, text mining was used to search for linkages among formally defined concepts using MeSH terms in PubMed records. For each PubMed record, MeSH descriptors were captured and listed (using a regular-expression-based script). Lists of diseases, symptoms, etc., were exact matched within the MeSH descriptors associated with each PubMed record. Moreover, a list of MeSH terms representing relevant clinical manifestations, including different types of pain, was searched for both within the PubMed entry-associated MeSH term list, as well as within the entire abstract text. This list of clinical signs was determined by veterinary experts with experience in renal diseases in cats and dogs. These were meant to be broad enough to capture interesting associations, yet focused on symptoms which are more likely to be observed in companion animals, rather than clinical tests (often measured later in the disease). Our approach assumed that any MeSH terms linked to a given publication would likely have a positive association with the other MeSH terms associated with that publication. Negative associations were less likely, and while this was still a possibility, our previous assessment of the methodology [33] suggested this would be negligible.

The list of terms used to search MeSH for clinical signs and pain types developed for this study can be found in Table S1; all other lists have been previously published [33,34]. Fisher’s exact test was applied in R [36] to test for independence between MeSH terms identified (in each of the categories) in human vs. cat publications and in human vs. dog publications. The total number of MeSH terms per category was used as the background. Venn diagrams of the MeSH terms captured among the three analysed species for each MeSH category were generated (Figure S1) using the ggVennDiagram package in R [37,38].

### 2.3. Network Analysis

Network analysis was caried out using Cytoscape [39,40,41]. Three types of MeSH terms, namely disease, pain, and clinical signs (symptoms), were used. In brief, a network was generated such that each MeSH term was represented as a node, and an edge between two nodes represented at least one co-occurrence of the two terms within the same PubMed abstracts. Node types and node and edge counts of co-occurrences were used to set the layout of the networks, colouring, and sizing. Nodes of interest with first-degree neighbours were used to create sub-networks. The matrices used to generate the networks are available in Supplementary data (Supplementary File S2).

## 3. Results

**Figure 1:**
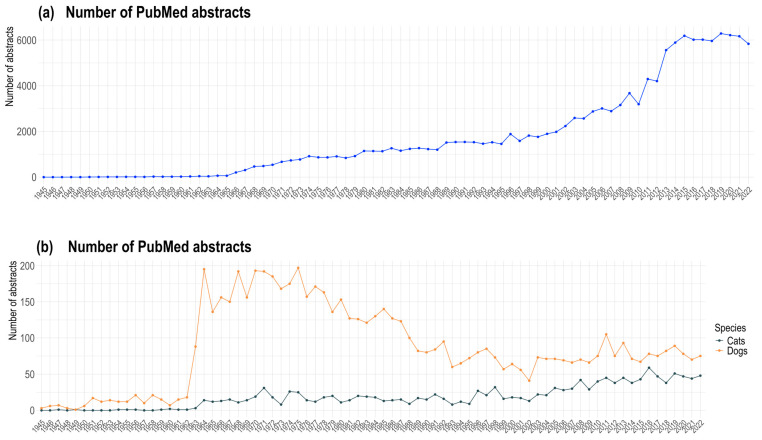
CKD and renal disease-related publications over time (in years) in (**a**) humans and (**b**) cats and dogs.

In total, our searches identified 138,343, 1458, and 6687 PubMed records related to CKD in humans and to renal disease more generally in cats and dogs, respectively. Since the number of publications directly related to CKD in cats and dogs was limited, we expanded our inclusion criteria to cover all renal disease; however, in humans, we limited our search to CKD specifically (see Methods). Articles’ publication periods spanned between 1945 and April 2023. Reports increased for all three species analysed in the 1960s (Figure 1). In humans (Figure 1a), published CKD research peaked between 2015 and 2022, reaching ~6000 abstracts per year. In cats, a continuous increase in publications was observed, reaching a maximum in 2016 (n = 59); in dogs, after the peak between 1964 and 1975, a decline in publications on CKD was found, with almost a 2-fold decrease in recent years compared to the peak decade within the analysed corpus (Figure 1b).

**Figure 2:**
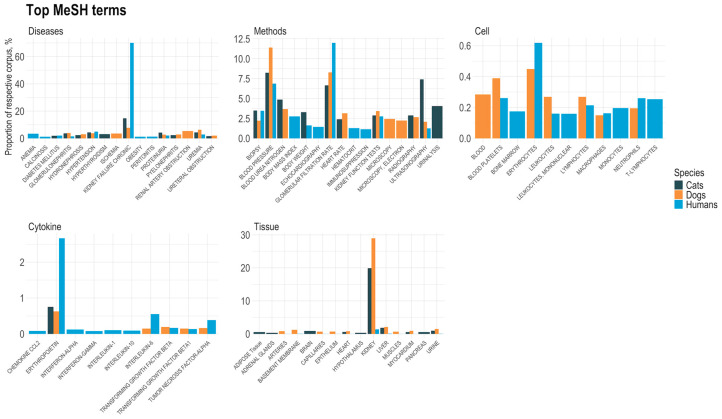
Top MeSH term associations in each category (diseases, clinical signs, clinical methods, cell types, cytokines, and tissue types) and per species (humans, cats, and dogs) in CKD- and renal disease-related PubMed records.

The MeSH terms that were searched for were grouped into categories and included a total of 67 cell-, 86 cytokine-, 8695 diseases-, 802 methods-, and 116 tissue-MeSH terms. Additionally, there were 28 MeSH terms related to clinical signs. Diseases was the top MeSH category within the analysed corpus of publications (187, 338, and 880 unique hits for cats, dogs, and humans, respectively) followed by research methods (154, 261 and 608 hits for cats, dogs, and humans, respectively). Cytokines was the less presented category, with 7 terms identified in the literature regarding cats, 21 in that regarding dogs, and 69 in that regarding humans (Table 2). In the tissue category, 42% of the MeSH terms overlapped across the three species, followed by the cell category with 32% of the MeSH terms being in common (Supplementary File S2). The top 10 (with at least 10 publications per species) MeSH associations (or fewer than 10 MeSH associations if there were <10 publications) found in each category (diseases, diagnostic and research methods, cytokines, cell types, and tissue types,) and per species related PubMed records are presented in Figure 2. Within the analysed corpus of publications found in PubMed, chronic kidney failure was the most prevalent disease term (70% in human literature; 15% in cats and 8% in dogs), followed by hypertension and anaemia in humans, hypertension and uraemia in cats, and uraemia and renal artery obstruction in dogs (Figure 2).

Most research and clinical methods in cats and dogs focused on blood pressure (120 and 762 publications, respectively), while in humans, this was the second most frequent (n = 9498), with the first being glomerular filtration rate (n = 16,600). Associations with different cell types and cytokines were much less frequent in the relative PubMed records (less than 1% for cells and 3% for cytokines), compared to clinical signs, diseases, and methods terms. In accordance with human subjects within the literature, mentions of erythrocytes were also the most prevalent in dogs (856 and 30 publications, respectively). Interestingly, associations with neutrophils and T-lymphocytes (around 350 publications for each) were found in humans, but none were found in dogs. On the contrary, the terms blood cells (n = 19) and leucocytes (n = 18) were present only in dog-related studies. For humans, dogs, and cats, erythropoietin (EPO) was the most prevalent cytokine (3685, 42 and 11 publications, respectively). Other cytokine terms were held in common for human and dog studies in the top 10 MeSH associations list, namely interleukin 6, transforming growth factor beta, transforming growth factor beta-1, and tumour necrosis factor-alpha, with all except tumour necrosis factor-alpha also found in the cat literature but in far fewer publications.

### 3.1. Clinical Signs

**Figure 3:**
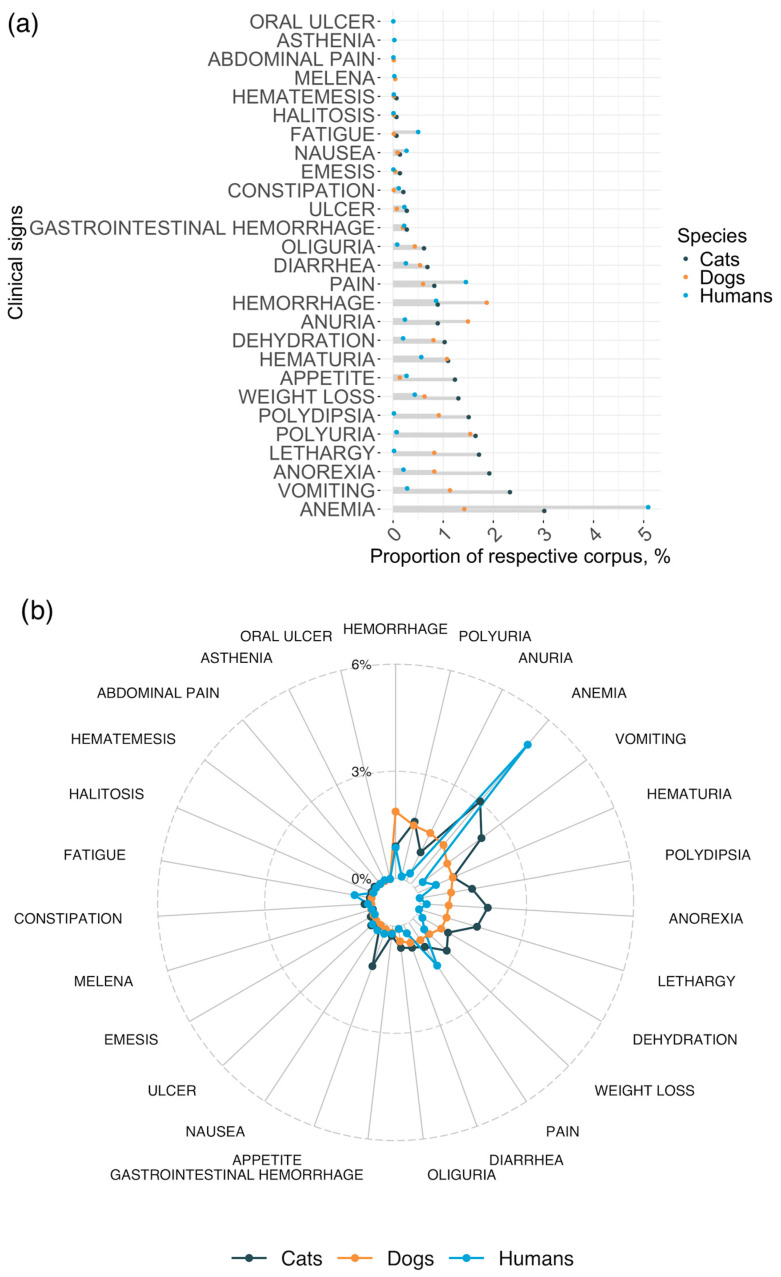

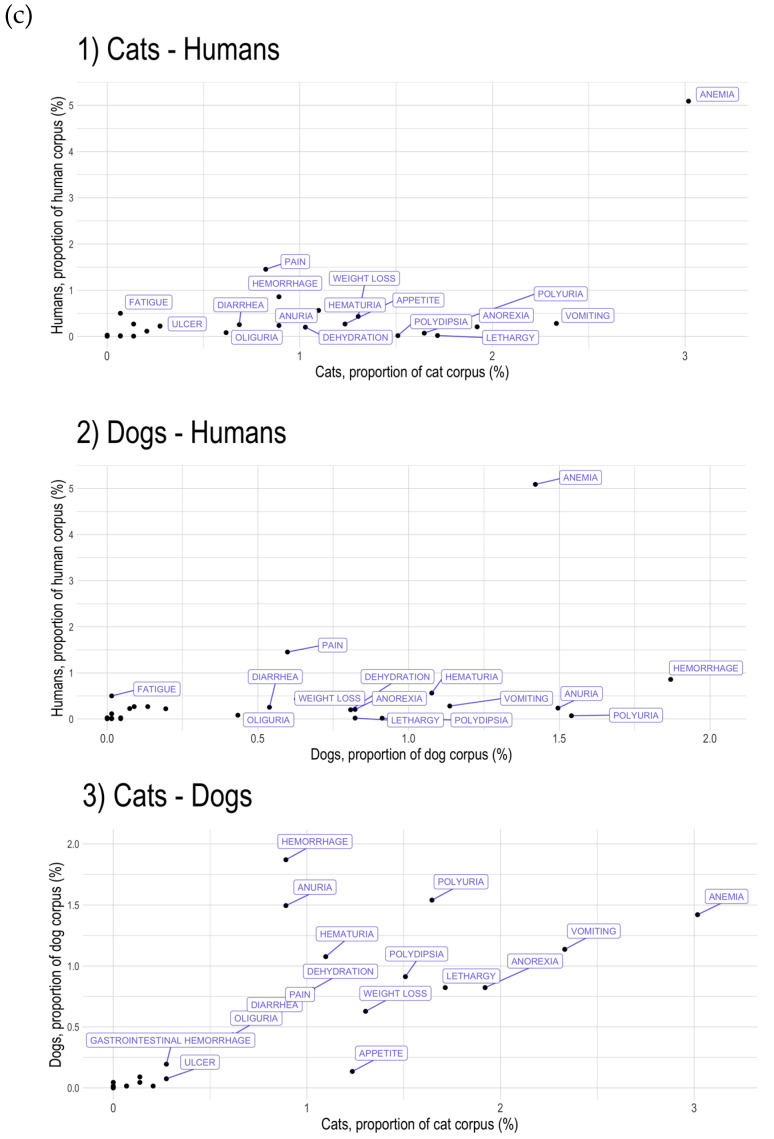
Forest plot denoting the proportions of each clinical sign MeSH term in the three corpuses (**a**), radar plot presenting the relative proportions of the different clinical signs across the three species (**b**,**c**), relative counts of associations of clinical signs in the human-, cat-, and dog-related PubMed corpuses. In (**c**), each dot represents a clinical sign, with placement along the x or y axes determined by the proportion of that clinical sign in the respective corpus.

MeSH terms within the clinical signs category resulted in non-significant differences between humans and cats or dogs (*p*-values of 0.18 and 0.11 for humans–cats and humans–dogs in Fisher’s exact test, respectively; Table 2). Figure 3 summarises the associations of clinical signs in the human-, cat-, and dog-related PubMed corpuses. In total, 17,164 out of the 146,488 analysed PubMed records (combined across all three species) referred to at least one of the various clinical signs and symptoms of CKD (15,857, 987, and 320 in humans, dogs, and cats, respectively). Of the complete list of 28 clinical signs searched for in the analysed corpus of publications, 23 were found across cats, dogs, and humans, while abdominal pain and melena (black sticky stool) were found in both dogs and humans (but not cats) and oral ulcer and asthenia were present only in humans. Anaemia was the clinical sign mentioned more frequently in humans (n = 7042) and cats (n = 44) and was the fourth most frequent in dogs (n = 95) (Figure 3). Apart from anaemia, vomiting was another symptom in common in all three species and in relative high frequency in all, with 389 records in humans, 76 in dogs, and 34 in cats. In dogs, haemorrhage was the most frequent clinical sign (n = 125), followed by polyuria (n = 103) and anuria (n = 100). Haemorrhage was also present in the human literature (n = 1188) and in cats (n = 13). Comparing cats and dogs, polyuria, lethargy, polydipsia, and anorexia were also reported.

### 3.2. Pain in CKD

**Figure 4:**
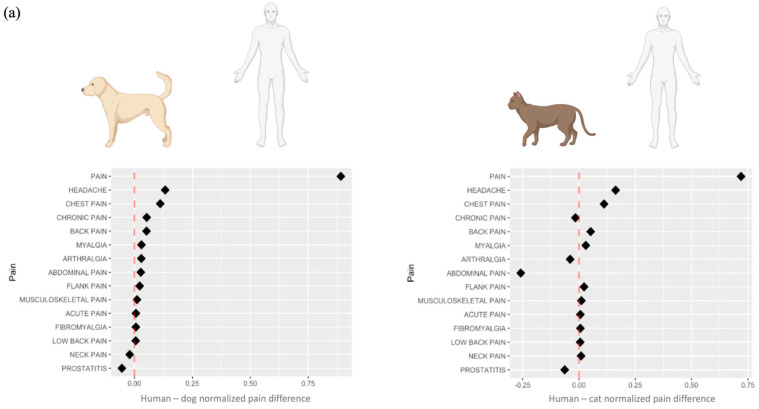

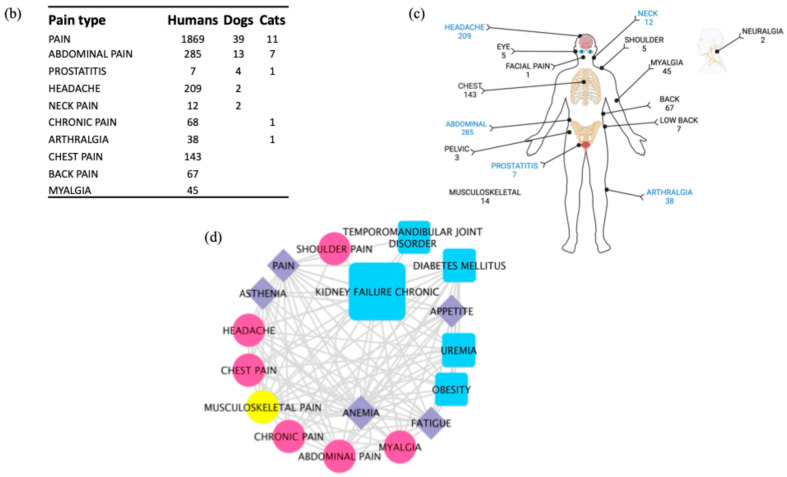
Pain terms in CKD- and renal disease-associated PubMed records. (**a**) Relative association of pain terms between humans and dogs or humans and cats. Points to the rights of the dashed red line have a higher frequency in human-associated publications as compared to dogs or cats. Points to the left of the dashed line represent pain terms that are proportionally higher in dog- or cat-related publications as compared to humans. (**b**) Top 10 pain terms and their absolute counts in the human, dog, and cat corpuses. (**c**) Different pain terms associated with human CKD-related publications, by anatomical location (created with BioRender.com); in blue—pain terms shared with cats and/or dogs. (**d**) Network analysis showing all interactions between disease (blue), clinical sign (purple), and pain (pink) MeSH terms in the human corpus; a sub-network was generated by selecting musculoskeletal pain and its directly associated terms within the network.

One interesting association revealed by our analysis was with the term “pain” in human CKD-related publications (n = 2010 in the human corpus). Thus, we further deepened our investigations into pain that may be related to renal disease and CKD. MeSH terms of different “types” of pain were searched for both within the PubMed entry-associated MeSH term list, as well as within the entire abstract text. Pain terms in CKD and renal disease-associated PubMed records are summarised in Figure 4. In total, 32 different pain terms were identified in human-related records, from which, seven were also found in either dogs or cats, or both (Figure 4a,b). More precisely, the MeSH term “pain” was the most prevalent MeSH term in all species, followed by abdominal pain. Headache and neck pain were found in both humans and dogs, while chronic pain and arthralgia were found in both humans and cats. Chest pain was reported only in the human literature and is ranked as the fourth most frequent pain term within the analysed corpus of publications. Different pain terms associated with human CKD-related publications are visualised by anatomical location in Figure 4c. It is important to note that for different types of pain, the number of records found associated with any of the terms in cats and in dogs is very low, limiting the conclusions that can be drawn directly from these data. However, the larger number of associations found in the human corpus of publications can be leveraged to direct potential areas for future research in cats and dogs.

A network of MeSH term (pain, clinical signs, and diseases) associations (co-occurrences) from human CKD-related publications was created. As an example, a sub-network was created for the term “musculoskeletal pain” (Figure 4d). This revealed associations with the symptoms of anaemia, fatigue, appetite, and weakness (asthenia), as well as other types of pain. This type of pain was also associated with diabetes, obesity, and uraemia in the human CKD corpus of publications. Other sub-networks revealed associations between different types of pain and disease and clinical signs. For example, in the context of CKD, abdominal pain was associated with peritonitis (n = 54), vomiting (n = 50), diarrhoea (n = 38), nausea (n = 30), and haemorrhage (n = 19), as well as ischemia (n = 18) and hypertension (n = 17).

## 4. Discussion

In this study, we examined possible links and parallels between chronic kidney disease in humans and renal disease and CKD in cats and dogs using data captured from MeSH term associations in related PubMed literature. The number of available publications related to CKD or renal disease greatly varied among species, with the number of available publications being far greater in the human-related literature. While publications related specifically to CKD in cats and dogs were fewer, we hypothesise that much can be learned from publications related to renal disease more generally in these species, but also, from CKD-related publications in humans where significant research has been carried out and published. By mining information from MeSH terms, we identified common features and trends (e.g., symptoms and diseases) in CKD shared across humans, cats, and dogs, as well as unique characteristics within species, which may warrant further investigation.

### 4.1. Recognising Pain in Animals

The prevalence of pain in humans with CKD has been estimated at ~50–70% [42,43], yet it may still be an undertreated clinical manifestation that is not given sufficient consideration [42,44]. Nevertheless, several studies have pointed to the association between pain and decreased quality of life in patients with advanced CKD [45,46,47,48,49,50]. Our network-based analysis of human MeSH terms specifically focused on pain revealed associations of CKD with musculoskeletal pain and abdominal pain (among others) that were further associated with a range of other clinical signs and diseases (Figure 4). As in human medicine, recognising and quantifying pain in animals is essential for diagnostic purposes and the effectiveness of the ensuing therapies. However, the detection of pain in animals remains challenging. In examining the co-occurrences of clinical signs between cats and dogs, vomiting was recorded in ~8% of the publications reporting symptoms in both species. Haemorrhage was found frequently in the human and dog literature (1188 and 125 out of 15,857 and 987 records in humans and dogs, respectively) but not emphasised in cats (n = 13 out of 320 records).

Assuming that common diseases across species share some common pathological pathways, the extrapolation of pain symptoms identified in humans may be beneficial for a more precise and early diagnosis in veterinary medicine. The limited number of available PubMed records related to pain (identified in the current study) in cats and dogs can be explained by the nature of the term, which may be difficult to determine by veterinarians. Alternatively, it may be, at least partially, due to lack of awareness of the importance of pain and pain assessment in renal disease in companion animals. The official guidelines of IRIS (http://www.iris-kidney.com/guidelines/guidelines_updates_2023.html; accessed on 28 February 2024) report that “Signs may include polyuria, polydipsia, weight loss, decreased appetite, lethargy, dehydration, vomiting, and bad breath”, currently not listing pain as a clinical sign for CKD in cats and dogs. Our results suggest that this may need reconsidering to highlight the importance and role of pain in this condition and to direct clinical efforts to detecting and treating it.

### 4.2. Cytokines Related to CKD

Cytokines are signalling proteins that play an important role in the immune system [51,52,53]. In all three species, EPO was the most prevalent cytokine, followed by interleukin-6 (in humans and dogs), tumour necrosis factor-α (in humans and dogs), transforming growth factor-β, and transforming growth factor-β1. EPO is a glycoprotein hormone that stimulates red blood cell production and is naturally produced by the peritubular cells of the kidney. It has been suggested that a progressive decline in endogenous EPO level is associated with the development of anaemia in CKD [54]. Moreover, it has been shown that EPO production in hypoxia is inhibited by members of inflammatory cytokines, namely interleukin-lα and interleukin-1 β, the transforming growth factor-β, and tumour necrosis factor-α [54,55,56]. Such associations with CKD, identified through the rich evidence base in human research, can highlight potential targets for future research in companion animals, where some concordant evidence is already emerging. Incorporating molecular biomarkers into diagnostic and prognostic approaches has demonstrated benefits in human CKD [28,57,58] and could similarly be applied, with further investigation, to CKD in companion animals.

### 4.3. Limitations

The text-mining approach used in our analysis to capture CKD-related associations from MeSH descriptors assumes that co-occurrences of the relative MeSH descriptors used within a PubMed record represent true and positive relationships. However, it should be noted that causality cannot be assumed from co-occurrence; neither can directionality. Further, any erroneous associations may be magnified where co-occurrences are present in only few records. However, as previously shown in [33], an extensive evaluation of the approach, comparing the human interpretation of MeSH-term associations from abstracts to that captured by the text-mining method, showed that at least 70% of co-occurrences of different types of MeSH entities (disease, cell type, or cytokine) signify true direct or indirect dependencies. Where numbers are sufficiently high, this generally identifies meaningful trends for a given topic. Nevertheless, this approach has not been previously tested in publications related to species other than humans, and it is possible that other elements which introduce “noise” exist. For example, some associations may be derived from publications where companion animals were used as an experimental model (rather than the patient) or where an association with renal disease is found in human subjects following an incident of a dog bite.

The lack of directionality and the type (nature) of the associations captured and the differentiation between early and late disease stages in the analysed corpus of publications is potentially a further limitation of the approach. For instance, anaemia and EPO were over-represented in the mined corpus of publications. However, anaemia is a common complication of CKD, since with CKD, the kidneys are damaged and thus EPO production decreases [54]. Hence, anaemia and EPO would not be able to serve as early predictors of CKD. Nevertheless, these co-occurrences may still hold valuable information, for example, where associations to date are anecdotal or non-existent in cats, but are supported by significant evidence in humans.

Despite the possible limitations, the ability to summarise hundreds of thousands of scientific records reported in different species with relative ease counterbalances any shortcomings by providing new insights though comparative analyses, especially for species where the research on CKD is limited. The identification of 32 different types of pain in CKD-related human literature, from which only 5 were in common between humans and cats and humans and dogs, justifies this. As the cost of multiplexed protein assays decreases, the ability to look at human disease-associated protein changes associated with a specific disease will inform work in companion animals. Thereby, we can compare and contrast kidney disease to a more sophisticated degree in future work, especially when multiplexed assays become available in companion animal studies.

Beyond the arguments for a One Health approach to disease and wellness, a reduction in the number of animals used in experiments for scientific purposes is a key objective. The EU Reference Laboratory for alternatives to animal testing reported that in 2019, ~70% of animals were used in basic, applied, and translational research in human and veterinary medicine (https://joint-research-centre.ec.europa.eu/eu-reference-laboratory-alternatives-animal-testing-eurl-ecvam/biomedical-research_en; accessed on 28 February 2024). In line with the 3R (refinement, reduction, and replacement) principles [59] for animals used for scientific purposes and an increased public interest in animal health and welfare, an extensive literature review can help to better organise experimental designs in biomedical research and thereby reduce the number of animals used in lab experiments for scientific purposes. For our purposes, improved understanding of human disease research to benefit veterinary medicine is a key goal served by the approach presented in this manuscript.

## 5. Conclusions

Using text mining, we presented results of comparative analysis for kidney disease in cats, dogs, and humans. Common, as well as unique clinical signs, including different types of pain, identified across the three species may pave the way for future research and treatment strategies in companion animals and help deepen our knowledge of CKD.

## Figures and Tables

**Table 1:** PubMed search strings used to query the database for renal disease and CKD-related publications.

	Humans	Cats	Dogs
**Search string**	((chronic renal insufficiencies[MeSH Terms]) OR (chronic renal insufficiency[MeSH Terms]) OR (Kidney Failure, Chronic[MeSH Terms])) AND ((Humans[MeSH Terms]) OR (Homo sapiens[MeSH Terms]))	((chronic renal insufficiencies[MeSH Terms]) OR (kidney diseases[MeSH Terms]) OR (chronic renal insufficiency[MeSH Terms]) OR (Kidney Failure, Chronic[MeSH Terms])) AND (felis catus[MeSH Terms])	((chronic renal insufficiencies[MeSH Terms]) OR (kidney diseases[MeSH Terms]) OR (chronic renal insufficiency[MeSH Terms]) OR (Kidney Failure, Chronic[MeSH Terms])) AND (canis familiaris[MeSH Terms])
**No. of resulting papers**	138,343	1458	6687

**Table 2:** Number of MeSH terms captured per category in each of the species and *p*-values of Fisher’s exact test.

MeSH Category	Total No. of MeSH Terms	No. of MeSH Terms Captured in Humans	No. of MeSH Terms Captured in Cats	No. of MeSH Terms Captured in Dogs
Disease	8695	880	187_172_ (1.01 × 10^−157^)	338_319_ (1.09 × 10^−311^)
Method	802	608	154_152_ (2.64 × 10^−18^)	261_251_ (3.44 × 10^−25^)
Cell	67	50	18_18_ (2 × 10^−3^)	23_23_ (2.00 × 10^−4^)
Cytokine	86	69	7_7_ (0.20)	21_21_ (5.00 × 10^−3^)
Tissue	116	100	43_43_ (3.00 × 10^−4^)	65_64_ (1.23 × 10^−5^)
Clinical sign	28	27	23_23_ (0.18)	25_25_ (0.11)

In subscript, the number of common MeSH terms between the cat–human corpus or dog–human corpus are shown; in parenthesis, the *p*-values of Fisher’s exact test for the cat–human or dog–human comparison are shown.

## Data Availability

Data are available as Supplementary data and in Supplementary File S2.

## Supplementary Materials

The following supporting information can be downloaded at https://www.mdpi.com/article/10.3390/ani14233349/s1. Supplementary File S1: Table S1: List of curated terms used to search MeSH for clinical signs and pain types developed for this study; Figure S1: Venn diagrams of all MeSH terms captured in human, cat, and dog corpuses of publications in each MeSH category. Supplementary File S2: Supplementary data: Matrices used to generate the networks.
