# Supplementary material for: Renal Disease in Cats and Dogs—Lessons Learned from Text-Mined Trends in Humans
Source: Animals (Basel). 2024 Nov 21;14(23):3349. doi: 10.3390/ani14233349 (PMC11639467; doi:10.3390/ani14233349)

Figure S1: Venndiagrams of all MeSH terms captured in human, cat and dog corpus of publications in each MeSH category.

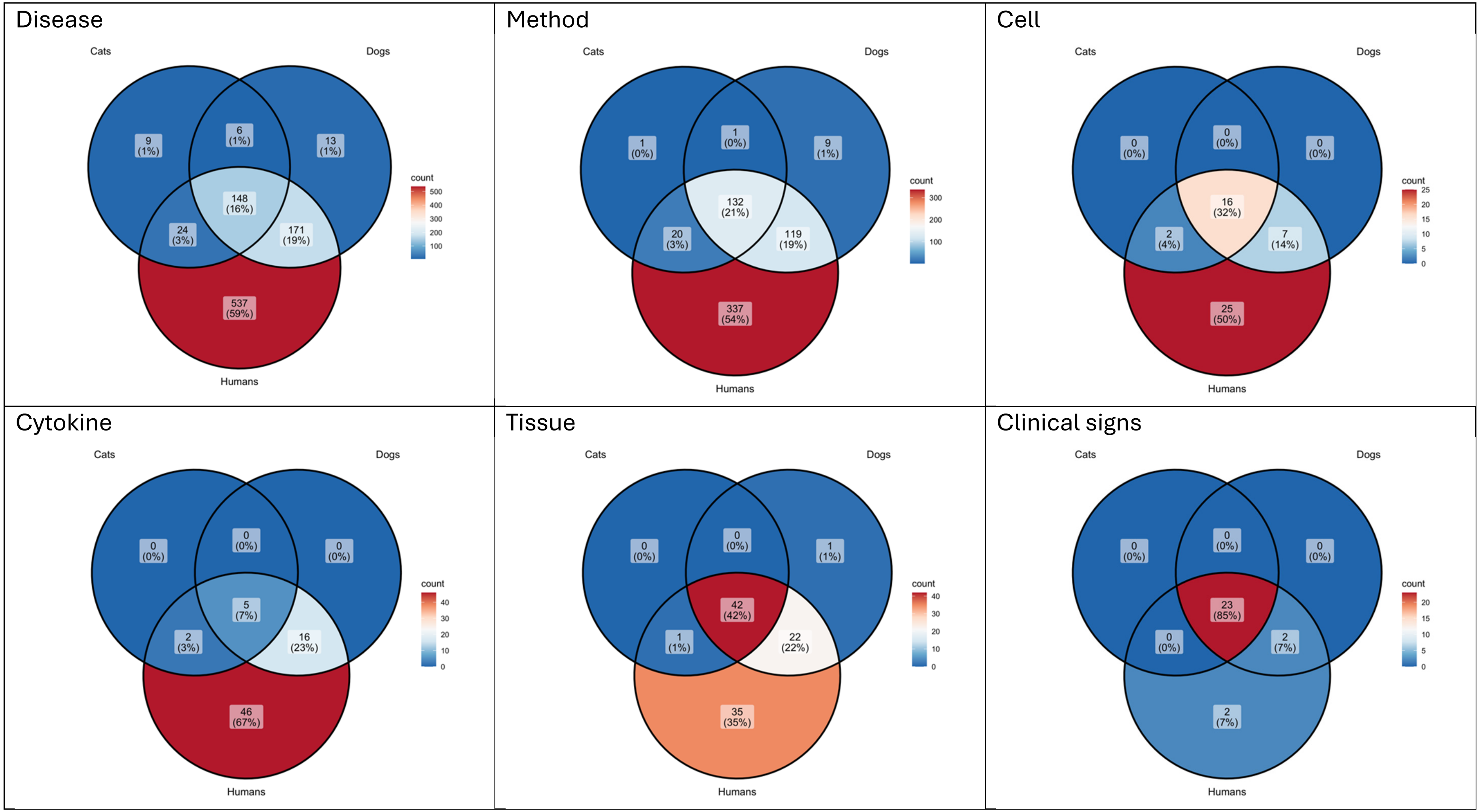

Supplement: Supplementary file 1 [file animals-14-03349-s001.zip › animals-3285634-supplementary/Supplementary_file_S1/Figure S1.pdf]
